# Drawing and the dynamic nature of living systems

**DOI:** 10.7554/eLife.46962

**Published:** 2019-03-27

**Authors:** Gemma Anderson, John Dupré, James G Wakefield

**Affiliations:** 1 Living Systems InstituteUniversity of ExeterExeterUnited Kingdom; 2 Egenis, The Centre for the Study of Life SciencesUniversity of ExeterExeterUnited Kingdom

**Keywords:** Philosophy of biology, Art, philosophy, cell division, mitosis, microtubule

## Abstract

Representing the dynamic nature of biological processes is a challenge. This article describes a collaborative project in which the authors – a philosopher of biology, an artist and a cell biologist – explore how best to represent the entire process of cell division in one connected image. This involved a series of group Drawing Labs, one-to-one sessions, and discussions between the authors. The drawings generated during the collaboration were then reviewed by four experts in cell division. We propose that such an approach has value, both in communicating the dynamic nature of biological processes and in generating new insights and hypotheses that can be tested by artists and scientists.

Biological theory often struggles to reflect the dynamic nature of living phenomena (Dupré and Nicholson, 2018). This difficulty is reflected in, and exacerbated by, the challenges of visually representing dynamic biological process. Cell division is one such process (Duncan and Wakefield, 2011). The majority of cell division is termed mitosis, where dynamic protein filaments (microtubules) coalesce to form a complex structure called the mitotic spindle. The spindle exerts physical force upon the duplicated chromosomes in the cell to segregate them into two equal complements. This is usually followed by cytokinesis, during which the cell membrane constricts to divide the cell into two new cells, each with one set of chromosomes.

The archetypal description of mitosis was communicated by Walther Flemming in a series of elegant drawings between 1878 and 1888 (Figure 1A; Paweletz, 2001). Recent advances in imaging have led to significant improvements in our understanding of this process (Figure 1B). However, despite these developments, 2D representations of mitosis and cell division remain virtually unchanged (Figure 1C).

**Figure 1.**
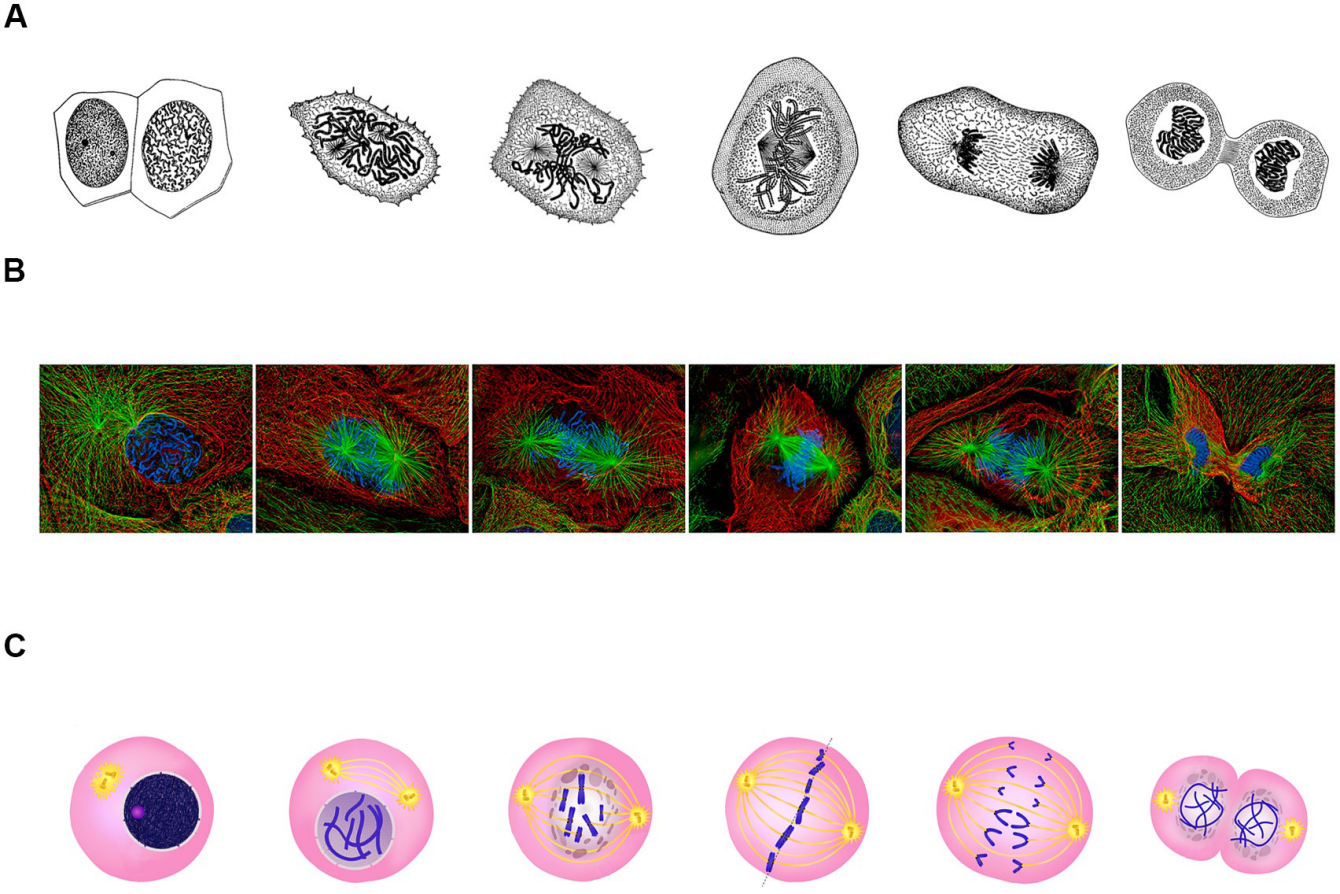
Classical representations of cell division. (**A**) Walther Flemming's 1888 drawings of eukaryotic mitosis (Image credit: adapted from Walther Flemming, CC0). (**B**) Confocal fluorescent microscopy images of newt lung cells during mitosis in culture (Image credit: Alexey Khodjakov, CC BY 4.0). (**C**) A diagram of the stages of cell division (Image credit: Ali Zifan, CC BY-SA 4.0).

Moreover, advances in imaging have resulted in a change in the role of the researcher: whereas cell biologists once used drawing to synthesise what they had seen in thousands of microscope images of cells, the power of imaging technology means that they now focus on measuring what they see. While this has had many advantages, we would argue that one downside of the decline of drawing is that it has eliminated a degree of exploratory imagination – and, therefore, a source of new ideas and hypotheses – from the scientific process.

In this article we describe 'Representing Biology as Process', a trans-disciplinary project in which an artist (Gemma Anderson), a cell biologist (James Wakefield) and a philosopher of biology (John Dupré) collaborated to develop new ways of using drawing to explore cell division. The purpose of the project was two-fold: to generate images that can convey the dynamic nature of cell division; and to provide new insights into this process.

In the first stages of the project Anderson and Wakefield worked together, with input from Dupré, to produce 2D images of cell division that attempted to represent the multi-dimensional nature of this process. The outcome was a series of images very different from those found in textbooks. Moreover, discussions within the project and feedback from external experts suggest that the new images can provide genuine insights into cell division.

## Drawing labs as a tool to explore pictorial representations of mitosis

We began the drawing process by undertaking a series of collaborative Drawing Labs focused on mitosis. The intention was for the artist to encourage scientists to use drawing to explore the scope and limits of their knowledge while simultaneously learning about cell division. These group activities reintroduced drawing into scientific practice in a supportive and challenging environment, promoting the refinement and development of ideas within an iterative loop between the artist and the scientists.

**Figure 2.**
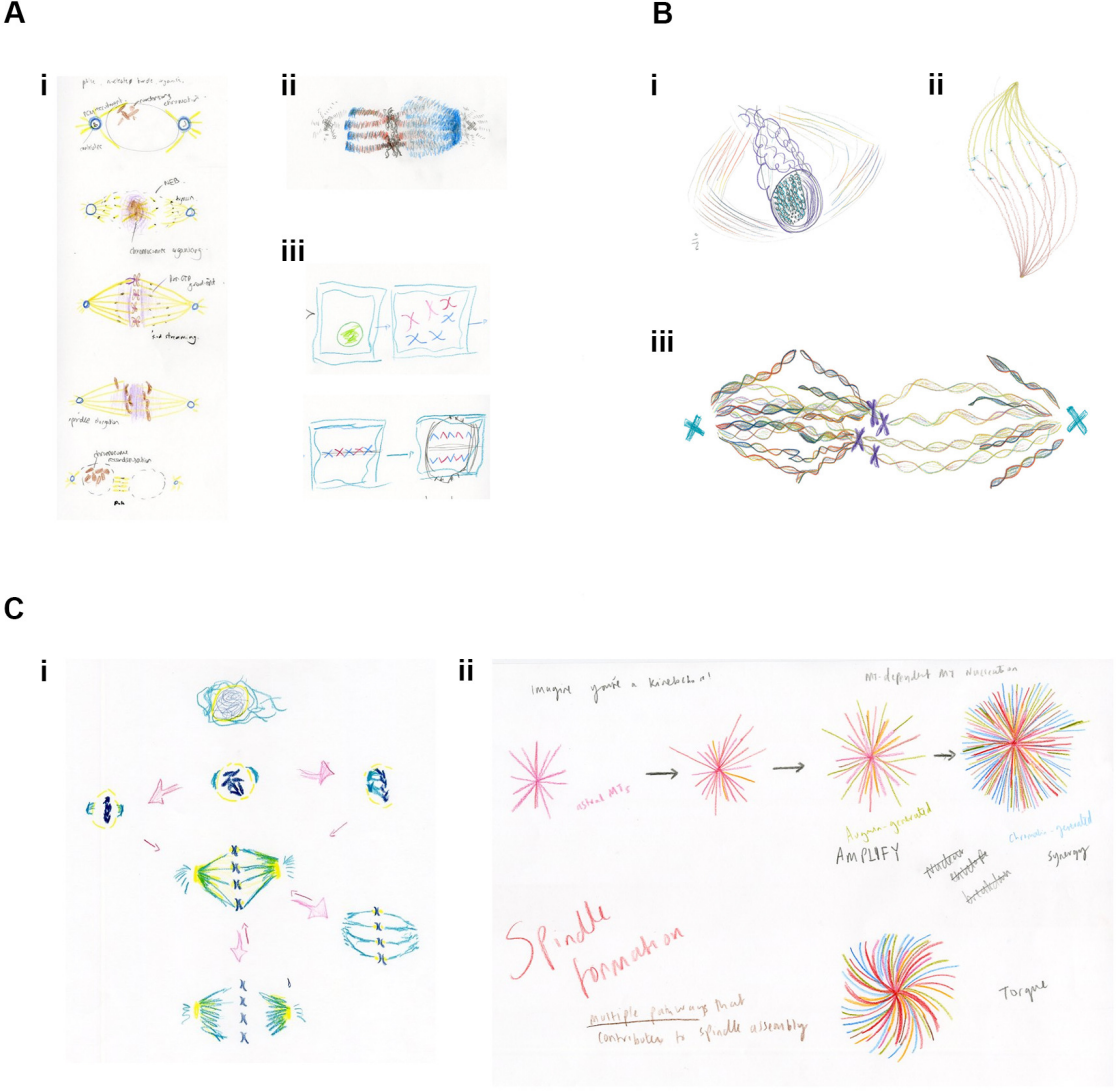
Examples of drawings from the Drawing Labs. (**A**) Results from session one. (**i**) PhD student drawing of stages of mitosis. (**ii**) Researcher drawing of mitotic spindle, emphasizing differences between kinetochore microtubules (left) and spindle microtubules (right). (**iii**) Researcher drawing of stages of mitosis. (**B**) Results from session two. (**i**) Researcher drawing, imagining the spindle from alternative angle. (**ii**) Anderson (artist) drawing mitotic spindle, emphasizing chirality. (**iii**) Researcher drawing of metaphase, emphasizing the helical nature of microtubules, emanating from the centrosome (right), and from both the centrosome and the chromosomes (left). (**C**) Results from session three. (**i**) Researcher drawing, with arrows reflecting the many possibilities associated with mitosis. (**ii**) PhD student drawing of mitotic microtubule generation. Words, colours and shapes combine to accentuate relationships in time and space and between the forces acting upon and within the spindle.

In the first session, a short visualisation exercise focusing on imagination and sense withdrawal aimed to turn the attention inwards and to build mental pictures through a kind of 'inverse vision' (Anderson, 2017; Anderson et al., 2015). The results, however, mostly revolved around classical, physical representations (Figure 2A). The second session aimed to increase the group's perception of the processes within mitosis in order to generate a consensus and to move towards producing a connected space-time image of mitosis that the artist could then transform into art. To guide the researchers, the artist asked the group to consider form patterns (or 'omnipresent morphologies'), such as the relationship between monomers and polymers. A recent article describing an intrinsic chirality within the mitotic spindle influenced some drawings (Novak et al., 2018), reflecting a more open, imaginative interpretation of spindle formation (Figure 2B).

The third session moved further towards a 'processual view' by introducing selected words and theoretical concepts as guides and catalysts for artistic vision. A list of 'mitosis verbs' generated by Wakefield and based on Anderson's previously published *Isomorphogenesis* approach (Supplementary file 1) was used, alongside a discussion of the works of Goethe, Waddington, Goodwin and Kauffman (von Goethe and Naydler, 1996; Waddington, 1957; Goodwin, 1963; Kauffman, 1996). It also included an introduction to concepts from dynamical systems theory, including the idea of flow systems. The group was invited to think about 'primitive' or archetypal forms, or to imagine a time slice within mitosis, along with the spatial possibilities that arise as each temporal 'snapshot' progresses. The group was also encouraged to think about the differences between mitosis in normal cells and mitosis in cancer cells. The resulting drawings, perhaps not surprisingly, tended to include arrows and additional descriptive words, similar to scientific figures (Figure 2C).

The first three sessions emphasised the difficulties that scientists had in breaking away from traditional, structure-based representations of mitosis, so we searched for analogies that would encourage more dynamic drawing. With choreographic principles and musical analogies as a guide, Anderson introduced the theme of the 'score' into the fourth session. The spatiotemporal interactions between DNA and microtubules during cell division are often described as a 'dance' (Yang and Yu, 2018; Klutstein and Cooper, 2014; Gough, 2011; Stukenberg and Foltz, 2010; Munro, 2007), while in choreography, music or art a score suggests a set of guides or cues that are interpreted by multiple elements (individuals, instruments) through time. Moreover, musical analogies have figured in several attempts to rethink biological ideas, such as the genome as a jazz score, or more general metaphors of life as music (Porta, 2003; Noble, 2008).

**Figure 3.**
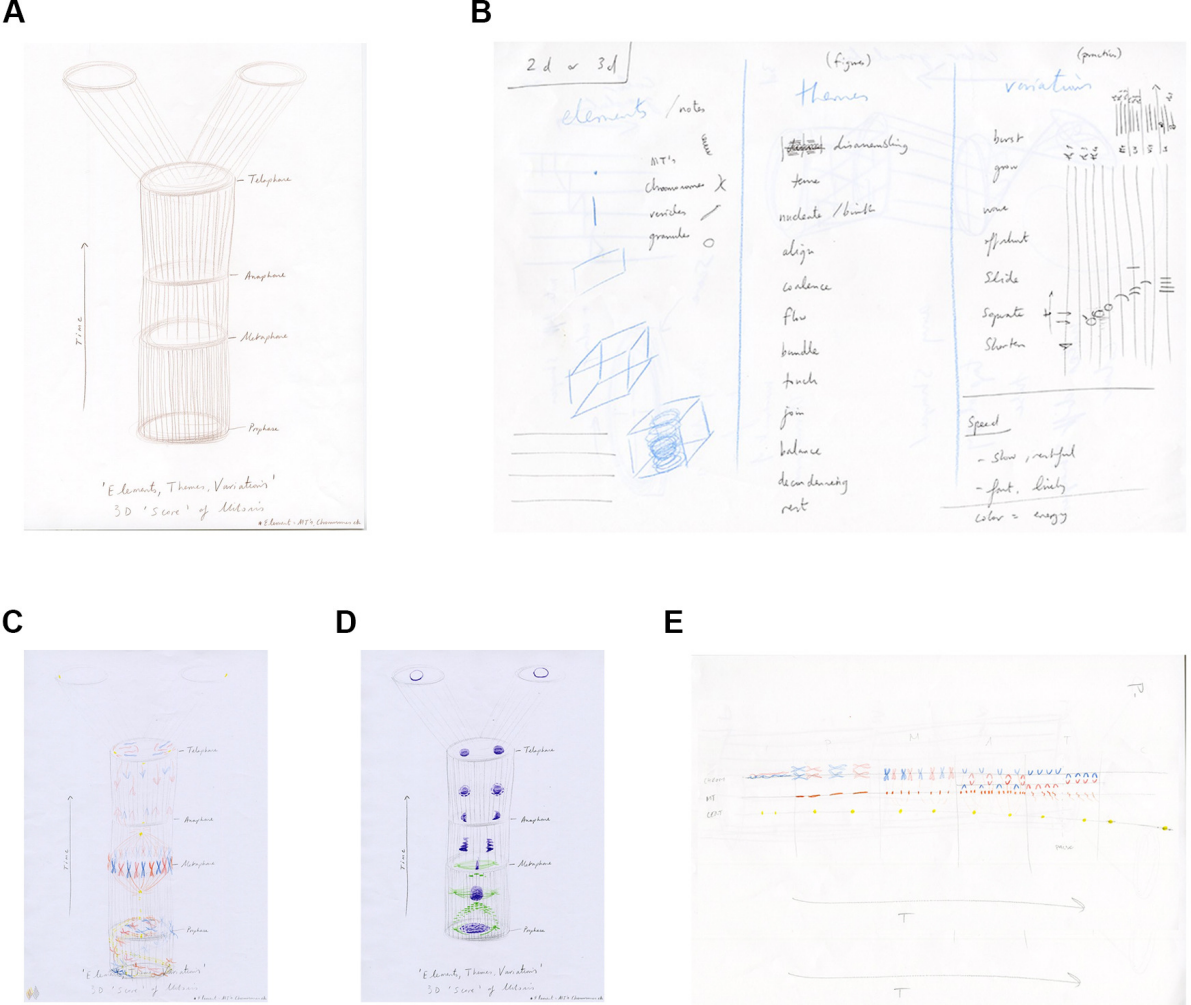
The score template and initial exploratory drawings. (**A**) Artist's drawing of a 4D (3D plus time) score template for session four. (**B**) Artist sketch of the contents for the flow system: elements of mitosis, choreographic and musical terms and verbs. (**C, D**) PhD student drawings of 'polyphony' of mitosis elements within the 4D score template. (**E**) PhD student drawing of the 2D score template: the decision of this student to rotate the template may have reflected their interest in classical music.

Anderson drew a 3D score in time (4D) to be read vertically (height=time) as a template structure for the group and invited them to imagine and draw the elements of mitosis as if a polyphony within the score (Figure 3A,B). It was noted that the score template was similar to a kymograph (a popular way of presenting spatial information over time; Figure 1; Hayward et al., 2014). The score template therefore provided a framework within which participants could explore and draw elements of mitosis to facilitate more exploratory and engaged interactions (Figure 3C–E).

## Refinements through interactions between artists and scientists

**Figure 4.**
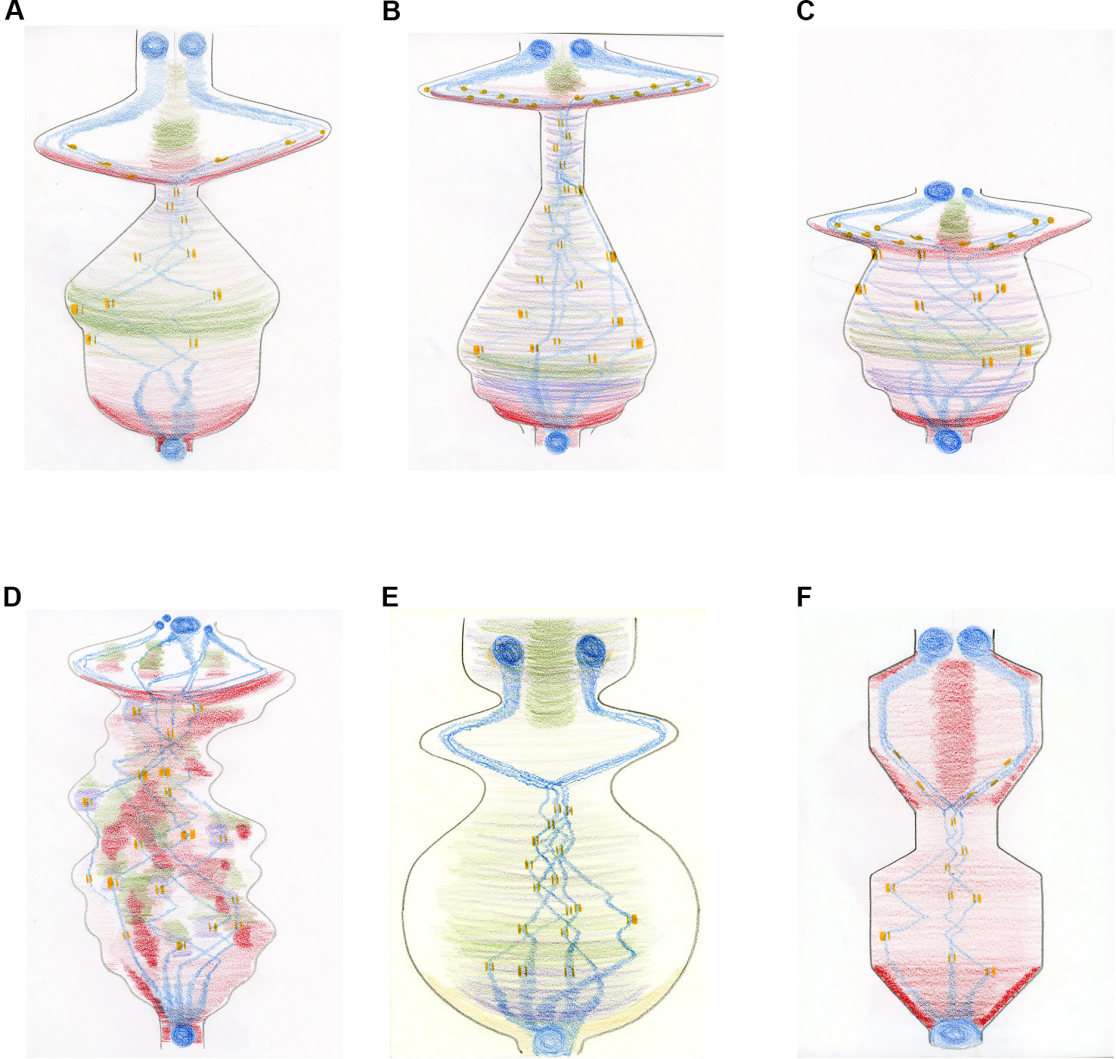
The mitosis score in different cell types. Creating a more 'processual image' by changing the shape of the score and depicting specific microtubule-generating pathways in different colours. The images describe the movement of chromosomes (blue) and kinetochores (which attach the spindle microtubules to the chromosomes; orange) during the different phases of mitosis: mitosis starts at the bottom of the drawing and ends at the top. The condensation and decondensation of chromosomes and the push/pull forces upon kinetochores are indicated through different shapes (circles and bars; where the forces applied to individual kinetochores within a pair are represented by the thickness of the bar). The generation of new microtubules by the centrosomes is shown in red, with chromosome-dependent microtubule generation in purple and augmin-dependent microtubule generation in green. (**A**) Mitosis in a *Drosophila* embryo over time. The overall shape directly corresponds to the sum of the spindle/microtubule-generating pathways and their interactions with chromosomes. As mitosis starts, there is a large burst of microtubule nucleation from the centrosomes, roughly coincidental with the condensation of chromosomes. A full mitotic spindle forms, supplemented by augmin-dependent microtubules. Chromosome congression happens quickly as the microtubule generating pathways reach steady state. A very short spindle assembly checkpoint is followed by the segregation of chromosomes, driven by microtubule depolymerization. A population of microtubules, originally generated by centrosomes and supplemented by augmin-generated microtubules, form the central spindle required to keep the decondensing chromosomes/reforming nuclei apart. (**B**) Mitosis in a human tissue culture cell. The same principles as (**A**) apply. The extended 'body' reflects the increased time needed to align chromosomes (23 pairs instead of the four pairs in *Drosophila*) and the increased time between metaphase and anaphase (about 20 minutes in humans, compared with about 1 minute in *Drosophila*). Chromatin-dependent microtubule generation is visible due to the extended time required for chromosome alignment. (**C**) Abnormal mitosis in a human cell lacking the spindle assembly checkpoint (shown by the truncated shape), which results in abnormal chromosome segregation and the generation of nuclei of different sizes. (**D**) Abnormal mitosis in a human cell with a defective spindle stability, which causes ongoing spindle rebooting and the production of an instable protein mass. (**E**) Mitosis in a plant cell. With centrosomes absent in higher plant cells, the formation of microtubules is facilitated predominantly by chromatin and by the nuclear envelope, amplified by augmin-dependent microtubules. (**F**) Mitosis in fission yeast demonstrating a closed mitosis and a bar-like mitotic spindle, generated purely from spindle pole body-nucleated microtubules. Both spindle formation and anaphase are intuited as 'ratchet-like' and measured, rather than explosive.

With this framework in place, Anderson and Wakefield met on four further occasions and created and revised new drawings to advance the image. Their explorations led them away from just drawing morphological 'objects' to creating a more processual image. As Wakefield’s research focuses on how different microtubule-generating pathways contribute to mitotic spindle formation in *Drosophila* embryos, we initially focused on replacing physical representations of microtubules. This was achieved by incorporating the 'inputs' of these pathways through sculpting the boundaries of the score and providing each microtubule-based input with a different colour. Physical representations of chromosomes were retained, alongside a representation of kinetochores and the forces acting upon them (Figure 4A). The same principles were applied to drawings of mitosis in a human tissue culture cell (Figure 4B), two dysregulated human cells (Figure 4C,D), plant cells and fission yeast (Figure 4E,F).

## Exploring dynamics within cell division

At the time of drawing the scores described in Figure 4, it was unclear what the overall sculpted shape related to, besides somehow including a dynamic perspective on the organisation of microtubules. A series of conversations among the whole team helped to define the overall changing shape of the image as being related to the energy inherent in the microtubule system(s) over time. This key reflection allowed the same principle to be applied to the chromosomes. Energy is required to both condense chromosomes at the start of mitosis and to decondense them following segregation, whilst additional input is needed to nucleate microtubules around the chromosomes and, through kinetochores, to assist their alignment. The score was then extended to encompass the entire process of cell division by incorporating the cell cortex (i.e., the forces acting on the cell membrane), where the rounding of the cell prior to mitosis, cytokinesis, and the reshaping of the cell following division, provided a corresponding energy-related shape.

**Figure 5.**
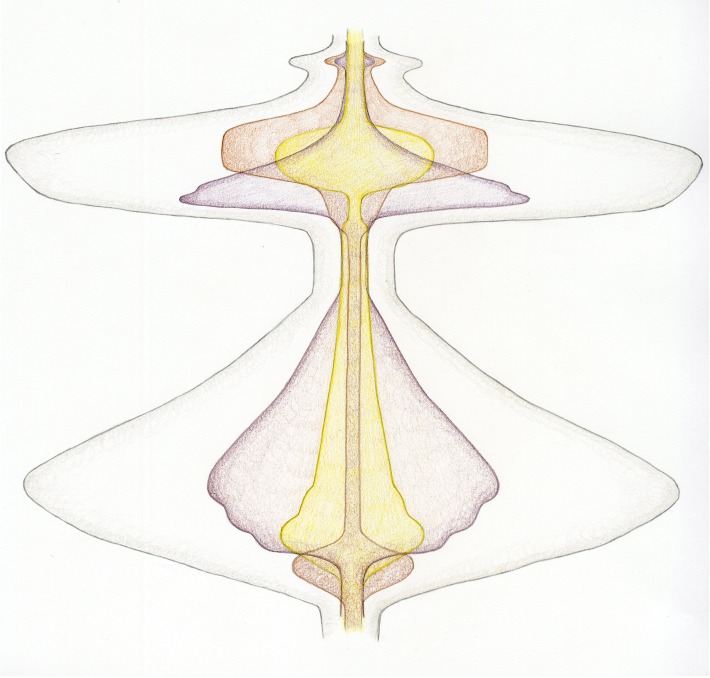
A new dynamic representation of cell division. Cell division, beginning at the transition from the G2 phase of the cell cycle to mitosis (bottom), and finishing after cytokinesis, when the cells divide (top). The energy related to the input of microtubule-generating pathways are now combined in purple, with chromosome-related processes in yellow and activities related to the cell cortex/membrane in brown. Just before mitosis starts, the cell actively rounds up. Then, during prophase, the microtubules nucleate, the chromosomes condense and are moved within the spindle. As chromosome alignment proceeds from prometaphase to metaphase, microtubules and chromosomes reach a steady state – hence the narrowing of the corresponding shapes. The activity of microtubules dramatically increases early in anaphase, which helps to segregate the chromosomes, which are just 'passive passengers'. By late anaphase, however, the decondensation of the chromosomes begins at the same time as the cortical acto-myosin contractile ring forms and contracts. Finally, cytokinesis itself occurs, requiring a small co-ordinated input from microtubules and the cell cortex. The outer grey shape represents the combined input of each activity described above – therefore corresponding to the overall energy/level of activity of cell division.

Further, unconventional colours (e.g., purple for microtubules, yellow for chromosomes, and brown for the cell cortex) were chosen to liberate the shapes from preconceived and widely-used nomenclature. Finally, by adding all the shapes together to form an overall outline, a full representation of the total process of cell division was generated (Figure 5).

## Testing the representation within the scientific community

To see whether the scores could be recognized as having a relationship to the physical entities, activities and sub-processes of cell division, we sent the drawings to four senior scientists in the field, each with over 25 years of experience. They were asked to describe their thoughts and feelings before being provided with a series of key words and, finally, explanations (see Supplementary file 2 for comments from expert observers, and Supplementary file 3 for a detailed explanation of the drawings). With only the final score (Figure 5) to look at, there was little to orient the experts. However, two asked whether the drawing was a kymograph, with one suggesting that "the purple or yellow represents separating chromosomes or spindle poles".

Providing the experts with initial keywords (kymograph and energy potential) was enough to elicit some very detailed responses that correctly identified the major elements of the pictures. Moreover, once the mitosis scores containing the physical representations of the blue chromosomes were shown, a majority of the experts was able to discern the key and relate the different pictures to various types of mitosis (Supplementary file 2). From these responses, we conclude that our new representation of cell division has value in conveying something of its dynamic nature, in relation to the key activities that contribute to it – at least to those familiar with the central concepts. We therefore believe that it could be a useful communicator of knowledge about processes in a pedagogic context.

## The processual representation of cell division as a hypothesis generator

One central aim of this project was to explore whether such dynamic representations could be collaboratively created with, and used by, scientists, drawing on their existing knowledge and intuition to generate new, testable hypotheses. Reflections by the researchers on the final score not only suggested analogies with other life processes (Supplementary file 4) but also brought to the fore a number of questions relating to the process of cell division.

For example, what sub-cellular activities outside of microtubules, DNA and the cell cortex are not represented by the current score? How would the shape change when other elements, such as the endomembrane system, are incorporated? Does the narrowing of the shape during metaphase simply reflect missing activities, such as the microtubule poleward flux (the continuous shortening and lengthening of the microtubules that does not affect the shape or size of the spindle, but that causes an energy flow from the centre of the spindle to the poles)? Or does the cell's energetic input into mitosis changes over time? If so, how close is the relationship between microtubule dynamics and mitochondria, the energy providers of the cell? Further, though mitochondria produce ATP, the energy source used for microtubule dynamics is GTP. How is this exchange in energy type accomplished? Where are the proteins that do this, and when do they do this?

These questions are new to the scientist involved in this project, even after 20 years of research and, as such, they provide a rich source of ideas for future experiments. Similarly, new questions are raised for artistic practice; for example, how should we imagine the repetition and variation of countless iterations of cell division? How can we represent time within a 2D framework? And how do we draw cell division as a process within processes, intersecting and interconnecting?

## Conclusion: drawing as a process-centred epistemology

Although the starting point for this project was an attempt to develop better representations of dynamic phenomena, a somewhat unexpected outcome has been the insight gained into the dynamic nature of the research process itself. Drawing shifts the focus from the image as product (an almost inevitable consequence of merely witnessing biological processes through various imaging devices) to the production of the image as an integral part of research. This activity, as described in preceding sections, can contribute to scientific insights and hypotheses. We suspect that these benefits might be even greater with drawing properly integrated into primary research rather than just exploring the representation of already established results. In the words of art historian Michael Podro: "We tend to consider images as subject matter only for visual scrutiny; as external – confronting the mind, as opposed to offering, like language, something in which the mind could participate. The problem for the defenders of images is therefore to show how they could – like language – be internal, open to the mind's participation, part of the mind's own thought and workings" (Podro, 2008).

Furthermore, artists have long drawn inspiration from scientific images, but here we see the artist co-creating images with scientists and thereby influencing the way that both understand the phenomena with which they engage. The process of drawing facilitates a move from reproducing what is seen towards imagining the shape of the biological process being studied (the ever-changing relationship between form and environment). It helps to envision the stages, dynamics and elements of which it is composed, and perhaps even to adopt an internal viewpoint of what it is like to be that phenomenon. The opportunity to explore and develop ideas, and the intellectual decisions about what to include and what to leave out of the image, give drawing its unique value as a way of knowing.

## Note

This Feature Article is part of the Philosophy of Biology collection.

## Additional files

Supplementary file 1. List of verbs describing mitosis.

Supplementary file 2. Comments from expert observers.

Supplementary file 3. Full explanation of the drawings.

Supplementary file 4. A cell biologist’s thoughts on the final drawing.
