# Supplementary material for: Drawing and the dynamic nature of living systems
Source: eLife. 2019 Mar 27;8:e46962. doi: 10.7554/eLife.46962 (PMC6436894; doi:10.7554/eLife.46962)
Supplement: Supplementary file 1. [file elife-46962-supp1.pdf]

tense

go!

burst (MTs)

grow (MTs)

expand/shrink (MTs)

nucleate/birth (MTs)

wave (MTs)

explore (MTs)

probe (MTs)

speckle (MTs)

offshoot (MTs)

align (MTs)

bundle (MTs)

slide (MTs)

coalesce (MTs)

focus (MTs)

flux (MTs)

flow (MTs)

mirror (MTs)

condense (chromosomes)

inhale (chromosomes)

tight (chromosomes)

touch (MTs and chromosomes)

kiss (MTs and chromosomes)

join (MTs and chromosomes)

co-ordinate (MTs and chromosomes)

dance (MTs and chromosomes)

align (MTs and chromosomes)

balance (MTs and chromosomes)

separate (MTs and chromosomes)

climax (MTs and chromosomes)

expend (MTs and chromosomes)

cleave (chromosomes)

shorten (MTs)

echo (MTs)

final pulse (MTs)

exhale (chromosomes)

relax (chromosomes)

decondense (chromosomes)

satisfied

rest
