# Supplementary material for: Drawing and the dynamic nature of living systems
Source: eLife. 2019 Mar 27;8:e46962. doi: 10.7554/eLife.46962 (PMC6436894; doi:10.7554/eLife.46962)
Supplement: Supplementary file 2. [file elife-46962-supp2.pdf]

Envelope 1: a new representation of cell division

This drawing represents an "archetypal" cell division. It is a representation and not supposed to be accurate in its parameters, but we believe the general trends that give rise to shapes in the drawing are "know-able", especially to those who have studied this process.

We would really like you to spend some time, in a relaxed setting (ideally not at work), looking and thinking about this image. How could it encode information about key processes associated with mitosis and cell division? What could the shapes and colours represent? How is the full process over time expressed in the drawing? What feelings does it evoke? Does it look like anything else (scientific or not)? Do "things" associated with mitosis and cell division appear?

Write down your impressions, thoughts and conclusions. These could vary from the very scientific, through to an ethereal stream of consciousness! (Remember, back before science was science, it was natural philosophy - and there was a much more holistic view of life and life-giving processes).

Don't worry if you cannot "get" any relationships between cell division and the drawing. We've never tried this before and the picture is the result of many months of discussion and drawing – it might be you need more implicit information to link what you see to cell division.

Once you have written down all you want, open Envelope 2

Comments on the drawing in Envelope 1 (continue overleaf if needed)

I notice that I am looking for similarities to what I know as "cell division", i.e. the classic microscope images of a mitotic spindle with separating sister chromatids, followed by cytokinesis. However, I do not see many similarities at first glance.

1st impression based on the grey lines and holding drawing with the text "drawing 1" in top right corner = Ingressing cell membrane, at least 3 ingressing furrows. Top one looks like a midbody with a midbody ring.

2nd impression: Butterfly and symmetry →

Continued comments on the drawing in Envelope 1

The drawing is almost perfectly symmetrical on the y axis. This suggests to me that the purple or yellow represents separating chromosomes or spindle poles. what is the brown colour representing?

Is the drawing some sort of kymograph of one round of cell division, followed by a 2nd rand of cell division of more cells (hence larger size of grey?)? If so, then why is the shape of the brown color, so different from the top?

Is the upper part a cell division, and the lower part S phase /G2 (duplication of DNA and centrosomes? kinetochores brown?

Third impression: Holding drawing in a different position (text "drawing 1" in bottom right corner) when looking from right to left (from "ingression 1" to "ingression 2") and from left to right (from "ingression 3" to "ingression 2") then in both cases, brown widens first, then yellow, and the purple. Two separate cells dividing?

Envelope 2: additional cues

Below are some key words that will help you draw some more insight into what the drawing represents (or will reinforce the insights you've already had):

KYMOGRAPH (UPWARDS)

ENERGY POTENTIAL OVER TIME

3 PROCESSES/THINGS RELATED TO CELL DIVISION, ENCOMPASSED WITHIN AN OVERALL REPRESENTATION

Now, repeat the process of looking and thinking, using the questions from Envelope 1 as a guide. Again, write down your impressions, thoughts and conclusions. Make sure you do this under the heading of Envelope 2, so we can see how the words have affected your understanding.

Continued comments on the drawing in Envelope 1 (having been given the above cues)
I am sticking to the kymograph. Although, I interpreted the kymograph downwards instead of upwards. Looking from that perspective I interpret the drawing as follows: prometaphase (relatively long phase) where chromosomes congress/align on the mitotic spindle (unsure whether yellow = spindle and purple are chromosomes, or the other way around?). Narrow part = metaphase, where chromosomes are aligned, which is followed by anaphase where sisters separate and by telophase (reformation of nucleus), and cyto-kinesis represented by some sort of midbody structure. Is there a chromosome lagging

Continued comments

behind in the midbody ?

Brown = nuclear envelope ?

purple = spindle ? poles ?

yellow = chromosomes ?

Alternatively option 3 : 3 processes / things related to cell division, encompassed within an overall representation ...

Process 1 : nuclear envelope breakdown and reformation

Process 2 : chromosome congression / alignment and segregation

Process 3 : cytokinesis.

Envelope 3: six drawings of mitosis

Envelope 3 contains a series of images from earlier incarnations of the final image. At that time, we were looking at mitosis (in the absence of cytokinesis), and were mixing "***things***" that maintained a shape and colour more concretely related to their biology and classical drawings, with the more "***process***"-based energy/potential of the system type of shape.

Take some more time, now, to discern how the different colours and shapes might relate to processes or subprocesses that occur during mitosis. One particular "thing" over time that represents a key element in mitosis might stand out. This should provide a reference point for you to explore the others.

Once you think you have worked out "the code", relate the different drawings to one another. What sort of mitosis is being represented in each them? Do the differences "ring true" to what you know about mitosis? As before, write down your thoughts.

Comments on the six drawings in Envelope 3 (continue overleaf if needed)

Drawing 4: Defective mitotic checkpoint.
→ no metaphase, unequal sister chromatid separation in anaphase.
→ Two new nuclei of different size

Blue = nucleus or space that the chromosomes take in? ~~chr~~ chromosomes lose confinement when entering mitosis (NEB). Blue lines represent chromosome movements

Red = ~~actin~~ actin?

green = tubulin / spindle?

Drawing 5: mitotic delay induced by a spindle poison (colchicine?)

Long prometaphase, no alignment of chromosomes
→ slippage from mitosis → one nucleus with several micronuclei

Continued comments on the six drawings in Envelope 3

"Floppy" cell shape.

Drawing 2 : "normal mitosis"

Blue : space that chromosomes take in,
chromosome tracks / movements.

Red : cell rounding ? Actin cytoskeleton ?

green : spindle ?

Relative short metaphase

Drawing 3 : Longer prometa and metaphase than ~~[illegible]~~ compared to drawing 2. More chromosomes that need to align ? Shorter prophase (red) ?

Drawing 6 : Too little actin ? Too little cortical rigidity that will ~~res~~ resist spindle forces / elongation ?

why are chromosomes not visible in anaphase ?

Drawing 7 : Too much ~~actin~~ actin ? Too much cortical rigidity ?

EXPERT OBSERVER 2

Comments to the drawing in Envelope 1

If I had seen the image without knowing that it was a representation of mitosis, I would have thought it was an image of a flower or of some rare (or imaginary) animal able to fly in either in the air or underwater.

I looked at the image several times before guessing its possible meaning. It is clearly a symmetrical mirror image with respect to an ideal perpendicular central axis. The symmetrical protrusions on the side of this axis could be mitotic structures but I cannot figure out what they are. It is possible that the process is represented over time like a kymograph but I cannot recognize the sequence of the events, consistent with my inability to recognize the colored structures.

The feeling evoked by the mage is of something that is struggling to become two but it is still in the middle of the process, so that it is unclear whether it will succeed.

Envelope 2: additional cues

I am glad that drawing 1 is indeed a sort of kymograph. However, even with the additional clues, I cannot figure out the biological meanings of the colored structures of the drawing.

Sorry, but I know very little about energy potential variation during cell division. I can only guess that energy potential is maximal in prometaphase cells and minimal at the end of mitosis (in telophase nuclei).

Envelope 3: six drawing of mitosis

Drawing 2: starting from the bottom I can see reed structures at both sides of a blue sphere (the nucleus) that could be centrioles. These structures are connected with red stuff that is likely to correspond to spindle microtubules nucleated by the centrosomes. Moving upward I can see that chromosome condense and the kinetochores become evident (yellow vertical lines; but why one line is thicker than the other?) and prometaphase movements occur. The chromosome then congress in a metaphase plate an anaphase occurs, followed by chromosome decondensation and formation of two daughter nuclei. However, the nature of the green material is unclear. Based on drawing 6, where there is not red material, I suggest that it could consist of MTs nucleated by (or in the vicinity) of kinetochores.

Drawing 3: This drawing differs from the previous one for the absence of “centrioles” and for a higher number of kinetochores.

Drawing 4: Here the chromosome fail to congress in a metaphase plate and segregation is abnormal leading to the formation of two daughter nuclei of different sizes.

Drawing 5: Here both spindle formation and chromosome segregation are disrupted.

Drawing 6: This cell lacks centrosome-nucleated MTs (red stuff), and only exhibit green MTs. Chromosome congression and segregation appear to be normal.

Drawing 7: This drawing has a peculiar shape compared with the others as it consists of two polygons of similar sizes. In addition, this cell only exhibit centrosome-nucleated red MTs and lacks green MTs. However, chromosome alignment and segregation appear to be normal

General comments on drawings 2-7

If my hypothesis is correct, namely that the green material are MTs nucleated by or near the kinetochores, then drawing number 6 could represent an animal cell devoid of centrosomes or a plant cells.

In drawing 2, I can see a pair of structures at both sides of the nucleus, each appearing as a pair of orthogonally arranged centrioles. However, in all other drawings with red MTs these structures are absent. Thus, either the paired structures are not centrioles (the most likely hypothesis), or you forgot to depict them in the other drawings with red MTs.

Recognizing the different types of cell depicted in the drawings is not easy. For example cells 2 and 3 appear to divide normally but differ in the number of chromosomes. However, none of these numbers correspond to the chromosome number of a well-known organism. In drawing 4, the chromosomes fail to form a metaphase plate and segregate abnormally even if the spindle structures appear to be regular, suggesting a possible defect in the spindle checkpoint. The cell depicted in drawing 5 is a mess with strong defects in spindle formation and chromosome segregation. As I mentioned earlier, drawing number 6 could be a plant cells, while I have no idea about the cell depicted in drawing 7. The only thing I can say about this cell is that it lacks of MTs nucleated by the kinetochores/chromatin.

Envelope 4: Summary

I have just a few notes about the drawings. First, I would use brighter colors in both drawing 1 and the other drawings. Specifically I would clearly distinguish between centrosome-, kinetochore/chromatin- and augmin-dependent MTs. If colors are too pale it is difficult to distinguish among the different types of MTs. For example, I did not note the purple kinetochore/chromatin-dependent MTs.

This is particularly evident in in drawing 6 where purple MTs are obscured by the mass of green MTs (are you sure that the amount of MTs generated by the augmin pathway is so preponderant on that of chromatin-dependent MTs?)

Second, I did not recognize the Drosophila cells because I was expected to see 8 kinetochores; moreover, in the same cell the anaphase chromosomes were fewer than 4 per half spindle. I understand that it is and artistic representation but, if you publish the drawings, you should indicate that the cell shows only the haploid complement.

Third, I am not sure that the representation of prometaphase kinetochores is correct, namely I do not understand why the thick bar is invariably oriented towards the cell poles.

I like the idea of using your representation of mitosis as tool to generate biological hypotheses. However, at the moment I have no idea on how to do so. In my opinion, to begin to explore this possibility you should generate drawings of mitosis of cells of the same type lacking a specific spindle component such as centrosomes, augmin, proteins necessary for kinetochore- driven MT growth, essential SAC proteins, and so on. Perhaps, based on these drawing, you will be able to make predictions on the mitotic phenotypes of cells missing other essential spindle components. In addition, based on this analysis, you might be able to recognize the primary defects responsible for mitotic disturbances caused by mutation or chemical treatments.

<u>EXPERT OBSERVER 3</u>

Drawing 1: Envelope 1

My first impression is not of mitosis at all but rather a crest for some type of fantasy movie character (thinking Narnia, Harry Potter, or some other type of fantasy movie) because I see golden colors, which are regal, and the general shape makes me think of something with it arms spread (at the top) and the royal cloak spread at the bottom.

If I try to look at the picture in terms of mitosis- what do I see.

- The center part of the drawing (the narrow strip) makes me think either of the S. cerevisiae spindle or the midbody at late mitosis.
- If I would choose colors for the normal spindle parts- I would choose the purple color for the chromosomes because they spread beyond the borders of the other structures. I would choose the brown for the microtubules, but I don't really like this choice because they are too rigid in the lower part of the drawing. I don't know what I think about the yellow. In the top part of the image it reminds me of the nuclear envelope because of its round shape- but then the other colors are not within the yellow- so this does not make sense. In the bottom- I can't get away from the fact that it looks like a big nose with nostrils at the bottom (sorry). In this version of the image- I don't know what my conclusion is for the grey except for the plasma membrane.
- If I turn the drawing 90° to the right, then I think of it as a time lapse of asymmetric chromosome segregation where the black/grey line is the cell membrane and the middle of the image is the cytokinetic furrow forming. Then I view the purple as a kymograph of anaphase A and anaphase B with the two rates of chromosome separation- but this doesn't make sense with the curves at the widest part of the purple section.
- Overall- this image doesn't bring a strong impression of mitosis to me- even in the more artistic sense.

Envelope 2

Keyword 1: Kymograph- that one I got the first time around- only now I get the timeline from top to bottom in that the chromosomes are not aligned they get aligned and then they segregate- I got all of this for anaphase- but not prior to metaphase in the first version of the image

Keyword 2: Energy Potential over time. I didn't think about this at all the first time with the drawing- but with you telling me this I still have to think about which color is energy. My initial thought was purple because that is the energy for segregation- but as I think about it- it doesn't make sense because if the first part of the picture (at the top) represents prior to metaphase then you can't have all of the energy right before chromosome alignment. Likewise, in chromosome segregation- this would say that the energy is at the end of the process. So then I think that maybe the grey line is the energy because you need it to get the chromosomes

aligned and then to get them segregated. The brown does not make sense for the energy because it is too little throughout anaphase in the bottom. And the yellow- this can't be energy, but it can't be chromosomes either because they are not fully separated at the end.

Keyword 3: Processes/things Related to Cell Division, Encompassed within an overall presentation: To me this is pretty vague. What I think it is referring to is the concept that the drawing illustrates the overall process of mitosis within a single image rather than the stepwise drawings that we typically see or a time lapse movie of the process.

Envelope 3:

Drawing 2: Blue immediately brought me to two ideas. If I look at the top of the diagram I immediately think centrosomes that are together and then separate. But that makes no sense as I go to the bottom half of the figure where the blue are clearly the oscillations of the chromosomes with the yellow/orange being the paired sister kinetochores- which don't actually split. But wait- now I am looking from the bottom to the top and the blue can be the chromosomes which begin to align and then oscillate and then they get tigher. At the narrowest point in the process they split and then decondense into the two daughter nuclei. This is honestly the first time that I considered looking at the drawing from bottom to top rather than from top to bottom.

Drawing 3: In this drawing as well as in drawing 2- I don't get what the green is. Throughout these drawings I don't get a feeling for the spindle itself. It can't be the green because the green just appears in two places. But it can't be the red because it is not prominent enough as the chromosomes are aligning- although it is in two structures at segregation, which could represent the anaphase B spindle elongating as the chromosomes segregate and then the formation of the central spindle. Still- what is the green?

Drawing 4: This looks like a bad direction of the project because there is very little that gets organized. The chromosomes never align, the blue at the end are asymmetrical, the colored lines in the bottom half of the image are equal intensity throughout.

Drawing 5: I think the artist had a little too much wine today. I still see the background of the oscillating chromosomes, but the red and green blotches look like some kind of modern art, which I am not really a fan of.

Drawing 6: This is much more focused on the chromosomes- but we lose the kinetochores afte segregation. Also- what is the little bit of yellow/orange near the separated masses at the end. Here the DNA is clearly in two separate masses- but I kept thinking of the grey as the membrane- but it is not between the DNA masses.

Drawing 7: This looks like a bacteriophage because of the symmetry at the top and the bottom of the image and the distinct red material in the center of the top portion. IN this one I will put

blue chromosomes, yellow kinetochores and red microtubules. While this one is clearly more visual to my scientific side- it does not have the artistry of the other drawings.

If I go back and comment about the type of mitosis- I'm not 100% sure that I know what you mean about type of mitosis- developmental, organismal?:

Drawing 2: This is the drawing that confused me as to what the different elements were in the first place. The chromosomes are asymmetric in their alignment.

Drawing 3: I have the same comments as in drawing 2 and as I noted above in that I am not 100% sure of what green represents.

Drawing 4: These chromosomes don't form a clear metaphase plate- so is this supposed to be yeast?

Drawing 5: If I go with my color code that I decided from above then the red are MTs, which makes no sense. Also- what is the purple- is it a mini ran gradient around each kinetochore?

Drawing 6: This literally says that the kinetochores dissolve at anaphase- but I cannot readily think of a situation where this happens

Drawing 7: This could be mammalian mitosis with a late aligning chromosome. The only thing I don't get here is why the chromosomes come back toward each other in the end.

Envelope 4: Summary- AKA Learning how much I failed at this entire process

Drawing 2 Explanation- I never got the idea that green was augmin dependent MT nucleation. I think I would have been helped immensely by different color choices- for example- use red and pink so that I would make a connection that both represent MTs- I kept trying to assign them different structures because red and green are definitely opposites on the color spectrum and when you think about a traffic light- red and green means stop and go respectively.

Drawing 3 Explanation- the purple was too subtle in this drawing- I came up with this idea when I got to drawing 5. I just simply missed the increased time for alignment in 3 versus 2- but I think my focus was figuring out what the structures were.

Drawing 4 and 5- Abnormal Mitoses. I clearly got that something was aberrant in these because I pointed out that they don't really align visibly. And I was stuck on the red in drawing 5. Again- the time differences didn't register with me.

Drawing 6- Oh- I am being stupid- I should have gotten this with Sid Shaw right upstairs from me. But I guess I was thrown off also because of the lack of red throughout because I had the as my MTs- and not as centrosomal MTs- which of course don't exist in plants.

Drawing 7- I clearly did not get this- but I did get the symmetry of it and the clear central spindle.

Overall- I don't know how I really did- I left my comments in the order in which I wrote them so you could see my thought development. I was also very hindered by assigning only one color to MTs because this influenced my thoughts all along the way.

I am curious as to how my colleagues did.

EXPERT OBSERVER 4

Envelope 1

In trying to get the relationship between the drawing and cell division, my first challenge was trying to figure out the orientation. I wanted it to go from left to right, with the yellow representing the chromosomes condensing, segregating and decondensing. But then the rest of it didn't quite make sense. I didn't see cytokinesis or two daughter cells, and if it is a cell cycle then it should repeat – one should be able to connect the end back to the beginning. If the outside line is supposed to represent the cell edge, it isn't right. I suppose that some lines are meant to represent cyclin/CDK activity, but then it doesn't make sense going left to right.

Envelope 2

Kymographs – then the drawing makes more sense oriented vertically, perhaps the range of chromosome (kinetochore) positions is in purple. Maybe DNA replication is in red. Yellow is chromosome condensation?

Envelope 3

Drawings seem to be oriented down to up, which doesn't seem right.

Orange must be sister kinetochores, blue must be DNA, area represents space occupied during chromosome oscillations?

Looks a bit like the female reproductive tract.

Envelope 4

I guess my sense is that kymographs should go top to bottom.

I totally missed microtubules, which is crazy since that's what I work on. The emphasis on augmin-dependent microtubule nucleation seems a little bit arbitrary as many other factors are involved.

I quite like the aberrant mitoses and now see how the drawings can represent variations in the theme.
