# Supplementary material for: Drawing and the dynamic nature of living systems
Source: eLife. 2019 Mar 27;8:e46962. doi: 10.7554/eLife.46962 (PMC6436894; doi:10.7554/eLife.46962)
Supplement: Supplementary file 3. [file elife-46962-supp3.pdf]

SUMMARY: Explaining the Drawings in Envelope 3

DRAWING 2 – Mitosis in a Drosophila blastoderm embryo

Background: This was the drawing we created first and was the one that went through several iterations before getting to the final image. Through a series of "drawing labs" with the Wakefield group and one-to-one interactions between Gemma (the Artist) and James (the Cell Biologist) we tried to find a "common language" that could be put on a page in drawing form. We kept coming back to the concept of the "choreography" of mitosis – how the chromosomes and microtubules interact, with the analogy of the dance between the two. This prompted us to look closer into choreography in general.

"*A choreographic work is generally devised as a 'score' which can be danced, but it can also be interpreted through other media. Thinking about biological processes, through the conceptual framework of 'choreography', could help to draw mitosis in an intuitive way. The dancer and choreographer William Forsythe considers choreography* 'an organisational practice' *and says* 'there are probably innumerable approaches to choreography […] choreography doesn't always look like a dance [in fact], dances don't always have to be danced […] because they are composed of information, and seeing [these] phenomena as information is a revolution, because they contain worlds of possibility' *(Forsythe). In this way, we can think of drawings as 'Biochoreographic images', that provide a way of stilling time in order to* 'watch, see what we know' *(Forsythe). To open up new possibilities for making images in science and to ask 'what else can this scientific knowledge look like?' and to translate biological movement into something that resonates choreographically and visually."*

In addition to drawing, Gemma also plays the harp. The choreography within music is visualised as a score; the scaffold upon which individual notes combine in harmony to create a dynamic flow that communicates interpretive meaning to its intended audience. She realised that musical notation can be read not only horizontally (following conventions of western reading style) but also vertically (as with eastern conventions) and that, for a 3D process, a 3D score might be helpful. She therefore generated a picture of a vertical tube, with the outline of a score held within it. This immediately connected with James, who has previously thought about designing a piece of music where different elements of mitosis (different microtubule nucleating pathways, chromosomes etc) are each "played" as different instruments, varying in volume, pitch, timbre and speed, as the "theme" of spindle formation is developed. Further, aware that the 'score' has an arbitrary tube-like shape, Gemma prefaced the interaction by asking James '*what shape does the process body of mitosis take*?' These concepts ran through a session in which James, led by Gemma's questioning, generated the essence of DRAWING 2.

Explanation: Time runs from the bottom of the drawing to the top – analogous to a kymograph representation where t=0 is at the bottom of the page. Each individual colour represents a process, pathway or thing. Red represents centrosomal MT nucleation – which leads and makes up the majority of "activity", early in mitosis, followed by augmin-dependent MT generation (in green). Blue (chromosomes) and kinetochores (orange) have a far more "spatial" composition, with condensation/decondensation and the push/pull forces upon kinetochores shown quite explicitly, by shapes (circles and bars; where the forces applied to individual kinetochores within a pair are represented by the thickness of the bar). At the time

of generating the image, it wasn't clear what the overall shape of the "pot" related to, except that it seemed to somehow represent something dynamic in relation to the process. Further analytical investigation in sessions with John (the philosopher) helped to define it as ***a measure of the change in activity/potential of the system over time***.

In this image, the shape directly corresponds to the sum of the spindle/MT-generating pathways and their interactions with chromosomes. So, upon entry into mitosis (bottom of the pot), there is a large, and dramatic burst of MT nucleation from the centrosomes (red), roughly co-incidental with chromosome condensation. A bipolar spindle shape is achieved about the same time as both Augmin kicks-in and kinetochore-kMT interactions begin in earnest. Gradual chromosome congression happens quickly (only 4 chromosomes in *Drosophila*), with the mature spindle exhibiting decreasing change over time (tapering) as a stable metaphase plate is achieved. A very short SAC is followed by the dramatic segregation of chromosomes, driven by depolymerisation at centrosomes, as well as "Pacman" from the kinetochores. Once full segregation has occurred, "activity" decreases, but is still represented by central spindle dynamics, composed of MTs originally generated by centrosomes (i.e. overlap MTs), and supplemented by Augmin-generated MTs (green). Chromosome decondensation and nuclear reformation then occurs.

The same concepts and parameters described for DRAWING 2, apply for all the images in Envelope 3 (i.e. DRAWINGS 3-7). Given the focus on MTs and chromosomes, and the derivation of these subsequent drawings from concepts cognisant in the *Drosophila* syncytial blastoderm, there is no consideration of cortical or cell membrane "activities" or cytokinesis.

DRAWING 3 – Mitosis in a human tissue culture cell

Explanation: The major differences between the human cell mitosis represented in DRAWING 3 and the original *Drosophila* embryo mitosis, are (i), the visualisation of chromatin-dependent MT generation (in purple), (ii) a more extended "body" of the first half of the image, reflecting the increased time taken to align chromosomes (23 pairs instead of the 4 pairs in *Drosophila*) and (iii) the increased time between full congression of chromosomes and anaphase (~20 mins in humans and only ~1 min in *Drosophila*). Although chromatin-dependent MT generation does occur in *Drosophila* embryo mitosis, it is not normally visible as a distinct "activity", due to the pre-primed centrosomes and fast spindle formation, in comparison to in human cells. It was therefore deemed important to include it in this drawing, but not in DRAWING 2.

DRAWING 4 and 5 – Abnormal Mitoses relating to cancer tissue culture cells

Explanation: Gemma asked the question "*what would a cancer cell mitosis look like?*", which led to the generation of two distinct pictures, each concentrating on one abnormal aspect of mitosis that has been described for cancer cells. The mitosis in DRAWING 4 is the consequences of an inactive SAC. The MT generating pathways, and chromosome condensation and alignment, are not affected. However, in the absence of a SAC, the "pot" is truncated in the middle. This truncation results in abnormal chromosome segregation and, ultimately, the generation of nuclei of different sizes. In our retrospective analyses of this

drawing, it did have a "squat" and "uncompleted" feel to it, as if it had been robbed of itd full potential.

In contrast, the SAC remains active in DRAWING 5, but defects in bipolar spindle stability result in continual "re-booting", where individual MT nucleating pathways periodically contribute mass that, ultimately cannot maintain coherence. Thus, centrosomal or chromatin-derived MTs are followed by "bursts" of augmin-dependent MTs, but chromosomes continue to oscillate, with the overall "energy" shape of the pot is irregular and abnormal. For the purposes of the drawing, an anaphase occurs – though in the cell one would expect many hours of delay in a prometaphase-like state before an abortive exit from mitosis. This abortive anaphase results in aneuploidy and the generation of both macro and micronuclei, due to incorrect chromosome segregation towards multiple, unstable poles. Again, subsequent analyses certainly provoked feelings of "messiness", "chaos" and a lack of symmetry and beauty, normally equated with a biological process.

DRAWING 6 – Mitosis in a Plant cell

With centrosomes absent in higher plant cells, MT nucleation upon entry into mitosis is initially facilitated predominantly by condensed chromatin and by the nuclear envelope (shown combined in purple), amplified by Augmin-dependent MTs (green). These dynamics result in a different shaped "pot" to those where centrosomes are present, both during spindle formation/chromosome alignment and in anaphase (which is slower than in animal cells). A more robust central spindle relates to the formation of the cell plate though, as in previous pictures, no cortical, membrane or (in this case) cell wall dynamics are considered. Our reflections on the pot, once this drawing was generated, converged on the "motherly" and somewhat "nature-rich" bowl-like shape, which resonated as strangely "plant like"…..

DRAWING 7 – Mitosis in a fission yeast cell

With a closed mitosis, and a "bar-like" mitotic spindle, generated purely from SPB-nucleated MTs, the fission yeast mitosis feels distinctly "analogue". Both spindle formation and anaphase are "ratchet-like" and measured, rather than explosive. With little direct experience of mitosis in fission yeast by James, this is probably the most "intuitive" (and incorrect?) representation of mitosis of all the drawings. However, something about the linearity of the shape rings true about a "basic" mitosis.

SUMMARY: Explaining the Drawing in Envelope 1 – the archetypal cell division

As eluded to, this drawing is the culmination of the project, containing the concepts and representations described for the earlier images, but removing the spatial/shape/object information relating to chromosomes and kinetochores. The MT/spindle generating pathways are now combined and shown in purple, but remain essentially as in Pot 3 (human cell mitosis). The representation of the ***change in activity/potential of chromosome-related processes over time***, is now given its own form and represented in yellow. So, the first "bulge" corresponds to the energy required for chromosome condensation, followed by an increasingly "passive" role as the chromosomes are pushed and pulled. This is not, however, minimised to zero, and an otherwise symmetrical bulge is supplemented by chromosomal activity present at kinetochores, driving both correct kMT generation and kMT attachment. This activity is more prevalent earlier in mitosis than at metaphase. The minimal "input" from chromosomes occurs in the short time space of anaphase, where they behave as passengers, followed by a renewed input of energy, related to chromosome decondensation (essentially the mirror-image of the condensation bulge initiated at NEB).

In brown, we now include activities related to the cell cortex. So, proceeding NEB, or any centrosomal-MT nucleation, the cell rounds up (whether in culture, or – to a lesser extent, as shown here – in a 3D tissue). Once spherical (in a non-polarised cell), the cortex does not input substantially into spindle formation or chromosome congression. However, increased dyanamics of lipids at the cortex slightly proceeds anaphase MT movements, and is quickly followed by the reinforcement of actin dynamics that results in a contractile ring. Contraction itself is represented as a linear phase, followed by a lessening input as the cell "rests" prior to abscission. This terminal event of cell division is depicted as requiring a relatively small co-ordinated input from both the cortical and MT activities.

The grey shape, encapsulating the different colours, represents the combined input of each activity described above – therefore corresponding to the overall activity/potential of cell division.
