# Supplementary material for: Drawing and the dynamic nature of living systems
Source: eLife. 2019 Mar 27;8:e46962. doi: 10.7554/eLife.46962 (PMC6436894; doi:10.7554/eLife.46962)
Supplement: Supplementary file 4. [file elife-46962-supp4.pdf]

Hi Gemma,
Of course.
I've just written the email to some very well established mitosis academics asking if they're willing to participate, so hopefully that will produce some good results.

I've been quietly contemplating the final cell division drawing. I am finding it quite awe inspiring and full of fresh meaning. Whether this is meaning I am placing on it due to the human capacity to find connections, or whether there is a deeper sense in which it is "right", I don't know.

It has a poise and regality to it that seems somehow ethereal (Charlotte saw it and said "angelic"). You can see within it the biological, animal form - with the symmetry, the rhythm and the flow of the lines representing the arms and the torso. And once you have that, you notice that the centrepoint of activity in the lower half rests within the "womb". And what is that womb, biologically speaking? It's the DNA being held, cradle-like, by the mitotic spindle. So you have the essence of life - the biological universality of replicated nucleic acid, carrying the potential to render life through division - in a cradle-like organisation of fibres, cosseting it, protecting it and holding it. And then to anaphase, where the arms (literally) are pulling the two genomes apart, quickly but with a security and depth provided by the safety of the cortex.

This isn't stuff that we can write for a journal but it has, as I say, breathed new life into a process that I've studied for 20 years.

The are some other thoughts - more scientific - that I think we can put in the article discussion, though, that pertain to the "unused" space in metaphase. Is that space real? and is it interesting? Almost certainly some of the space is, in practice, filled with flux (the continuous de-polymerisation and re-polymerisation of microtubules that do not affect the shape or size of the spindle, but that cause an "energy flow" from the centre of the spindle to the poles). But is there something else to be gleaned? If the space does not equalise out to the edges, this means that, biologically, energy use during mitosis changes, depending on mitotic stage. Does this also mean that energy production changes? Cells that are unable to fully align their chromosomes due to spindle problems arrest in pro metaphase but keep trying to assemble a spindle that is stable enough - this rearrangement, instability and reorganisation can go on for many hours, suggesting the cell continuously generates more energy. So what produces the energy in cells? Mitochondria. Where are they in the cell - and in the diagram? They produce ATP, but the energy source used by microtubules is GTP. How is this exchange in energy type accomplished? Where and when are the proteins that do this?

So, if the "job" of the drawing is to stimulate thought - it's certainly achieved its objective!

James
